# International Committee on Systematics of Prokaryotes Subcommittee on the Taxonomy of Rhizobia and Agrobacteria Minutes of the closed meeting by videoconference, 6 July 2020

**DOI:** 10.1099/ijsem.0.004784

**Published:** 2021-05-06

**Authors:** Philippe de Lajudie, Seyed Abdollah Mousavi, J. Peter W. Young

**Affiliations:** ^1^​ IRD, University of Montpellier, CIRAD, INRAE, SupAgro, LSTM, Montpellier, France; ^2^​ Ecosystems and Environment Research Programme, University of Helsinki, Finland; ^3^​ Department of Biology, University of Turku, Finland; ^4^​ Department of Biology, University of York, York YO10 5DD, UK

**Keywords:** rhizobia, agrobacteria, International Subcommittee on Taxonomy

## Minute 1. call to order

The annual Subcommittee meeting by videoconference was called to order by Peter Young at 11.00 UTC on 6 July 2020.

## Minute 2. Record of attendance

**Present (22):** J. Peter W. Young (University of York, UK, chairperson), Philippe de Lajudie (IRD, Montpellier, France, secretary), Mitchell Andrews (Lincoln University, Canterbury, New Zealand), Julie Ardley (Murdoch University, Perth, Australia), Bertrand Eardly (Penn State Berks College, Reading, PA, USA), Nemanja Kuzmanović (Julius Kühn-Institut, Braunschweig, Germany), Florent Lassalle (Imperial College, St Mary’s Hospital, London, UK), Kristina Lindström and Seyed Abdollah Mousavi (University of Helsinki, Finland), Esperanza Martinez-Romero (UNAM, Cuernavaca, Mor., Mexico), Lionel Moulin (IRD, Montpellier, France), Xavier Nesme (INRA, University of Lyon I, Villeurbanne, France), Joanna Puławska (Research Institute of Horticulture, Skierniewice, Poland), Emma T. Steenkamp (University of Pretoria, South Africa), Tomasz Stępkowski (University of Life Sciences, Warsaw, Poland), Chang-Fu Tian (China Agricultural University, Beijing, China), Pablo Vinuesa (Centro de Ciencias Genómicas – UNAM, Cuernavaca, Mor., Mexico), Gehong Wei (Northwest A and F University, Yangling, Shaanxi, China), Anne Willems (University of Gent, Belgium) and Jerri Edson Zilli (Embrapa Agrobiologia, Seropédica, Rio de Janeiro, Brazil).

**Apologies for absence (2):** Alvaro Peix (Institute of Natural Resources and Agrobiology, Salamanca, Spain) and Ridha Mhamdi (Centre de Biotechnologie de Borj-Cédria, Hammam-lif, Tunisia).

## Minute 3. Subcommittee publications

The minutes of our previous meeting (2019) have been published [1].

## Minute 4. Membership issues

**4.1**. As pointed out in the minutes of our last meeting, the Statutes of the International Committee on Systematics of Prokaryotes have been revised [2]. We considered carefully article 6 detailing rules on the organization and missions of subcommittees. According to these, regular members (those who are members of a society affiliated to the International Union of Microbiological Societies (IUMS) and having voting rights in the administrative workings of the Subcommittee) are: J. Peter W. Young (Microbiology Society), Seyed Abdollah Mousavi (Iranian Society of Microbiology, ISM), Bertrand Eardly (American Society for Microbiology), Florent Lassalle (Microbiology Society), Kristina Lindström (Biobioseura), Esperanza Martinez-Romero (Asociación Mexicana de Microbiologia), Alvaro Peix (Spanish Society for Microbiology), Chang-Fu Tian (Chinese Society for Microbiology), Gehong Wei (Chinese Society for Microbiology), Anne Willems (Microbiology Society, Belgian Society for Microbiology), Jerri Edson Zilli (Brazilian Society of Microbiology) and Emma T. Steenkamp (South African Society for Microbiology, SASM). Co-opted members are: Mitchell Andrews, Julie Ardley, Nemanja Kuzmanović, Philippe de Lajudie, Ridha Mhamdi, Lionel Moulin, Xavier Nesme, Joanna Puławska, Tomasz Stępkowski and Pablo Vinuesa.

**4.2. Admissions, Resignations**. Mitchell Andrews announced his need to withdraw from the Subcommittee due to recent job change and lack of time to contribute to the sub-committee’s discussions anymore. The Subcommittee will miss him as a very useful and contributive member and, in the name of all members, we thank him for taking his commitment seriously and for all his contributions over the years – they have been genuinely important and much appreciated.

**4.3. Chairperson and Secretary elections**. Prof. J. Peter W. Young was reappointed as the Chairperson of the Subcommittee and Dr. Seyed Abdollah Mousavi (University of Helsinki, Finland) was appointed as Secretary to serve for a 3 year term starting on 1 September 2020. There was no opposition among members and regular members validated these nominations by their unanimous votes. Philippe de Lajudie, as the current secretary, will inform the secretary of International Committee on Systematics of Prokaryotes (ICSP) subcommittees of this resolution. The members of the Subcommittee wish to record their gratitude to Philippe for all his hard work and devotion to the role of Secretary over the past years. He has been an excellent Secretary and has done a great deal to promote the activities of the Subcommittee.

## Minute 5. Following up the progress in resolutions (Minutes 6, 7, 7.1, 7.2) of the previous (2019) meeting

**5.1**. As decided last year, some members edited information on *Rhizobium* and *Pararhizobium* on Wikipedia pages. All members are encouraged to contribute to maintain these general public pages as updated as possible. The Subcommittee blog (https://taxonomyagrorhizo.blogspot.com/) is now available and should also be used to increase interactions and visibility of our recommendations.

**5.2**. The species table hosted on the Subcommittee website (https://sites.google.com/view/taxonomyagrorhizo/), which lists type strains and links to existing genomic data cannot be maintained daily up-to-date, and this will likely become harder to do as the number of described and sequenced species grow. The LPSN (https://lpsn.dsmz.de/) and GCM (http://gcm.wdcm.org/typestrain/about.jsp) provide more up-to-date information on taxonomy and links to genomic data, so it will be more value for our effort to produce a complementary type of information. It has then been decided to turn the species table into something containing a more specialized type of information, complementary to that provided on the other sites, e.g. the interactions of rhizobia and agrobacteria with plants, including symbiotic/pathogenic aspects, host range, symbiovars/pathovars, plasmid types, etc. For agrobacterial plasmids, the new groupings proposed by Weisberg *et al.* [3] could be used. The recorded data would aim at reflecting the genotypic/phenotypic diversity observed within the species, rather than just metadata associated with the species type strain. A new template/frame table will be designed by a subgroup of members (L. M., F. L., R. M., S. A. M. and others) so it can later be populated by all members with relevant expertise.

## Minute 6. New species, since the last meeting

We commented that proposed names should attempt to best reflect the biology of the new taxa. For instance, we have doubts if, in the case of newly described genera, the suffix ‘*rhizobium*’ should be used as the root of the name when the bacterium is not present in the soil environment or is simply nonsymbiotic, like the recently proposed genus names *Georhizobium* [4] and *Pseudorhizobium* [5, 6]. Similarly, the species name *Agrobacterium fabacearum* was proposed for *Agrobacterium* genomic species G1 (5), as it has, by chance, been isolated as an endophyte in bean root nodules, but not specifically as a symbiont. In the past, *Agrobacterium* G1 strains were isolated from several plant types [7] and detected in soils associated with *Poaceae* rhizospheres [8]. The plant specificity suggested for the genomic species G1 by the name *fabacearum* is somehow misleading. As a matter of fact and at the same time, Velázquez *et al.* [9] published evidence that *A. tumefaciens* is the correct name for the *Agrobacterium* genomic species G1, solving the long-standing problem of confusing equivalence with *A. radiobacter *, which is the correct name for *Agrobacterium* G4. The name *A. tumefaciens* has precedence over *A. fabacearum* and thus should have priority as the valid name *for Agrobacterium* genomic species G1. In addition, these authors request that the type strain of *A. tumefaciens* be corrected from ATCC 23308^T^ back to ATCC 4720^T^, since the original type strain ATCC 4720^T^ was never lost and is currently available in several culture collections. The Subcommittee supports these changes. Table 1 lists the novel taxa.

**Table 1.** Novel taxa described since the last meeting of the Subcommittee The names indicated in inverted commas have been proposed in effective publications, but have not yet been validated by publication in the IJSEM (do not figure in a validation list).

Species and nomenclatural type strain	Origin	T-strain Genome NCBI assembly (RefSeq) accession	Symbiotic/ pathogenic genes	Planttests	Reference
***Agrobacterium***					
* Agrobacterium cavarae* RZME10^T^ (=CECT 9795^T^=LMG 31257^T^)	*Zea mays* L. root, Spain	GCF_004310285.1	–	–	[20]
* Agrobacterium fabacearum* CNPSo 675^T^ (=UMR 1457^T^=LMG 31642^T^)	Nodules of soybean and common bean in Brazil, Mexico, Ecuador and Mozambique	GCF_009649785.1	*vir*	Nod-	[21]
*** Allorhizobium ***					
*'Allorhizobium oryziradicis'* comb. nov. N19^T^ (=ACCC 19962^T^=KCTC 52413^T^)		GCF_001939045.1			[11, 22]
* Allorhizobium taibaishanense* comb. nov. CCNWSX 0483^T^ (=DSM 100021^T^=HAMBI 3214^T^)		GCF_001938985.1			[11]
* Allorhizobium terrae* CC-HIH110^T^ (=BCRC 80932^T^=JCM 31228^T^)	Paddy soil, Taiwan	GCF_004801395.1			[22]
*** Bradyrhizobium ***					
* Bradyrhizobium archetypum* WSM 1744^T^ (=CNPSo 4013^T^=LMG 31646^T^)	Root nodule of *Muelleranthus trifoliolatus,* Western Australia	GCF_013114835.1	*nodA*, *nifD*, *nifH*	Nod+	[23]
* Bradyrhizobium australiense* WSM 1791^T^ (=CNPSo 4014^T^=LMG 31647^T^)	Root nodule of *Indigofera* sp., Western Australia	GCF_013114825.1	*nodA*, *nifD*, *nifH*	Nod+	[23]
*'Bradyrhizobium campsiandrae'* UFLA01-1174^T^ (=INPA 394B^T^=LMG 10099^T^)	Root nodule of *Campsiandra laurilifolia*, Amazon rain forest flooded area, Brazil	GCF_014529705.1	*nodA*, *nifD*, *nifH*	Nod+	[24]
* Bradyrhizobium cosmicum* 58S1^T^ (=LMG 31545^T^=HAMBI 3725^T^)	Non-nodulating diazotroph originating from soybean nodule, Canada	GCF_007290395.1	*nifD*, *nifH*	Nod-	[25]
* Bradyrhizobium hipponense* aSej3^T^ (=DSM 108913^T^=LMG 31020^T^)	Root nodule of *Lupinus angustifolius*, Tunisia	GCF_008123965.1	*nodA*, *nifD*, *nifH*	Nod+	[26]
* Bradyrhizobium murdochi* WSM 1741^T^ (=CNPSo 4020^T^=LMG 31651^T^)	Root nodule of *Rhynchosia minima,* Western Australia	GCF_000472965.1	*nodA*, *nifD*, *nifH*	Nod+	[23]
*** Cupriavidus ***					
* Cupriavidus agavae* ASC-9842^T^ (=LMG 26414^T^=CIP 110327^T^)	*Agave* L. rhizosphere in northeast Mexico	GCF_004217045.1		L.+	[27]
*** Devosia ***					
* Devosia ginsengisoli* Gsoil 520^T^ (=KACC 19440^T^=LMG 30329^T^).	Ginseng cultivation soil	GCF_007859655.1			[28]
* Devosia indica* IO390501^T^ (IO390501^T^=JCM 32636^T^)	Sea water sample from the Indian Ocean	GCF_003056405.1			[29]
* Devosia marina* L53-10-65^T^ (=MCCC 1A05139^T^=KCTC 72888^T^)	Deep seawater, South China Sea	GCF_009758415.1			[30]
* Devosia subaequoris* HST3-14^T^ (=JCM 14206^T^=KCTC 12772^T^)	Beach sediment sample from Hwasun Beach in Jeju, Republic of Korea	GCF_014197055.1			[30]
*** Georhizobium ***					[4]
* Georhizobium profundi* WS11^T^ (=MCCC 1K03498^T^=KCTC 62439^T^)	Deep-sea sediment sample collected from the New Britain Trench	GCF_003952725.1			[4]
*** Mesorhizobium ***					
*'Mesorhizobium alexandrii'* Z1-4^T^ (=KCTC 72512^T^=CCTCC AB 2019101^T^)	Phycosphere microbiota of marine dinoflagellate *Alexandrium minutum*	GCF_004000235.1			[31]
*'Mesorhizobium rhizophilum'* YM1C-6-2^T^ (=CGMCC 1.15487^T^=DSM 101712^T^)	Rhizosphere of maize grown in northeast PR China	GCF_003666685.1			[32]
* Mesorhizobium terrae* NIBRBAC000500504^T^ (=KCTC 72278^T^=JCM 33432^T^)	Soil in Jangsu, Republic of Korea	GCF_008727715.1			[33]
*** Methylobacterium ***					
* Methylobacterium crusticola* MIMD6^T^ (=KCTC 52305^T^=MCCC 1K01311^T^)	Biological soil crusts, PR China	GCF_003574465.1			[34]
* Methylobacterium durans* 17SD2-17^T^ (= KCTC 52908^T^=NBRC 112876^T^)	Gamma-ray-irradiated soil, Republic of Korea	GCF_003173715.1			[35]
* Methylobacterium nonmethylotrophicum* 6 HR-1^T^ (=GDMCC 1.662^T^=KCTC 42760^T^)	Tungsten mine tailings, Jiangxi, PR China	GCF_004745635.1			[36]
*Methylobacterium planium* YIM 132548^T^ (=CGMCC 1.17323^T^=NBRC 114056^T^)	*Lepraria* sp. lichen collected from Yunnan, southwest PR China	GCF_008806345.1			[37]
* Methylobacterium symbioticum* SB0023/3^T^ (=CECT 9862^T^=PYCC 8351^T^)	Spores of the AMF *Glomus iranicum* var. *tenuihypharum*	GCF_902141845.1			[38]
*'Methylobacterium terrae'* 17Sr1-28^T^ (=KCTC 52904^T^=NBRC 112873^T^)	Gamma ray-irradiated soil	GCF_003173755.1			[39, 40]
* Methylobacterium terricola* 17Sr1-39^T^ (=KACC 52905T=NBRC 112874^T^)	Gamma ray-irradiated soil, Republic of Korea	GCF_006151805.1			[41]
*** Microvirga ***					
* Microvirga arsenatis* 3D203^T^ (=SYSU G3D203^T^=CGMCC 1.17691^T^)	Hot spring sediment, Tibet, western PR China	GCF_009910705.1			[42]
*'Microvirga calopogonii'* CCABU 65841T (=LMG 25488^T^=HAMBI 3033^T^)	Root nodule of *Calopogonium mucunoides*, Yunan, PR China	GCF_003347665.1	*nodA*, *nifH*	Nod-	[43]
'*Microvirga tunisiensis'* LmiM8^T^ (=CECT 9163^T^=LMG 29689^T^)	*Lupinus micranthus* and *L. luteus* grown in Northern Tunisia	GCF_009296195.1	*nodA*, *nifH*	Yes	[44]
*** Neorhizobium ***					
* Neorhizobium vignae* comb. nov. CCBAU 5176^T^ (=DSM 25378^T^=HAMBI 3039^T^)		GCF_000732195.1			[11]
***'Neopararhizobium'***					[11]
*'Neopararhizobium haloflavum'* comb. nov. KCTC 52582^T^ (=MCCC 1K03228^T^=XC0140^T^)		GCF_002750855.1			[11]
*** Ochrobactrum ***					
*'Ochrobactrum soli'* BO-7^T^ (=KACC 19676^T^=LMG 30809^T^)	Soil of cattle farm, in Seosan, Republic of Korea	GCF_003664555.1			[45]
* Ochrobactrum teleogrylli* LCB8^T^ (=KCTC 72031^T^=CGMCC 1.13984^T^)	Insect *Teleogryllus occipitalis,* deserted cropland, Shuangliu, Chengdu, PR China	GCF_006376685.1			[46]
*** Paraburkholderia ***					
* Paraburkholderia agricolaris* BaQS159^T^ (DBS0351125^T^=NCTC 14075^T^)	Internal symbionts of spores of *Dictyostelium discoideum*, eastern USA	GCF_009455635.1			[47]
'* Paraburkholderia atlantica *' CNPSo 3155^T^ (=ABIP 236^T^=LMG 31643^T^)	*Mimosa pudica* and *Phaseolus vulgaris* grown in soils of the Brazilian Atlantic Forest	GCF_009362785.1			[48]
*'Paraburkholderia bonniea'* BbQS859^T^ (=DBS0351127^T^=NCTC 14076^T^)	Internal symbionts of spores of *Dictyostelium discoideum*, eastern USA	GCF_009455625.1			[47]
* Paraburkholderia elongata* 5N^T^ (=DSM 110722^T^=LMG 31705^T^)	Hemlock forest soil	GCF_013177735.1			[49]
* Paraburkholderia flava* LD6^T^ (=KACC 21387^T^=JCM 33640^T^)	Forest soil sample in Suwon, Gyeonggi-do, Republic of Korea	GCF_004359985.1			[50]
'*Paraburkholderia franconis'* CNPSo 3157^T^ (=ABIP 241^T^=LMG 31644^T^)	*Mimosa pudica* a*nd Phaseolus vulgaris* grown in soils of the Brazilian Atlantic Forest	GCF_009362735.1			[48]
*'Paraburkholderia hayleyella'* BhQS11^T^ (=DBS0351126^T^=NCTC 14077^T^)	Internal symbionts of spores of *Dictyostelium discoideum*, eastern USA	GCF_009455685.1			[47]
*'Paraburkholderia lycopersici'* TNe-862^T^ (=LMG 26415^T^=CIP 110323^T^)	N2-fixing, rhizoplane of *Lycopersicon esculentum* Mill. var. Saladette, Mexico	GCF_900096975.1			[51]
* Paraburkholderia madseniana* RP11^T^ (=DSM 110123^T^=LMG 31517^T^)	Forest soil following enrichment with 4-Hydroxybenzoic acid	GCF_009690905.1			[49, 52]
*Paraburkholderia panacisoli* DCY113^T^ (= KCTC 52951^T^=JCM 32098^T^)	Ginseng cultivation soil in Gochang-gun, Republic of Korea	GCF_008369935.1			[53]
* Paraburkholderia solitsugae* 1N^T^ (=DSM 110721^T^=LMG 31704^T^)	Hemlock forest soil	na			[49]
*'Paraburkholderia youngii'* JPY169^T^ (=LMG 31411^T^=SARCC751^T^)	Brazilian and Mexican *Mimosa*	GCA_013366925.1			[54]
*** Pseudorhizobium ***					
*'Pseudorhizobium banfieldiae'* NT-26^T^ (=DSM 106348^T^=CFBP 8663^T^)	Arsenopyrite-containing rock, sub-surface goldmine, Northern Australia	GCF_000967425.1		Nod-	[5]
*'Pseudorhizobium flavum'* YW14^T^ (=DSM 102134^T^=CCTCC AB2013042^T^)	Organophosphorus insecticide-contaminated soil	GCF_902502825.2			[5]
*'Pseudorhizobium marinum'* MGL06^T^ (=DSM 106576^T^=MCCC 1A00836^T^)	Seawater, surface of the South China Sea	GCF_000705355.1			[5]
*'Pseudorhizobium endolithicum'* JC140^T^ (=DSM 104972^T^=HAMBI 2447^T^)	Sand rock matrix	GCF_902153245.1			[5]
*'Pseudorhizobium halotolerans'* AB21^T^ (=DSM 105041^T^=KEMC 224-056^T^)	Chloroethylene-contaminated soil, Suwon, Republic of Korea	GCF_902153235.1			[5]
*** Rhizobium ***					
*'Rhizobium desertarenae'* ADMK78^T^ (=MCC 3400^T^=KACC 21383^T^)	Saline desert soil, Rann of Kachchh, India	GCF_005860795.2			[55]
*'Rhizobium deserti'* SPY-1^T^ (=ACCC 61627^T^=JCM 33732^T^)	Biological soil crusts, Mu Us Sandy Land, PR China	GCF_004358025.1			[56]
* Rhizobium dioscoreae* S-93^T^ (=NBRC 114257^T^=DSM 110498^T^)	Endophytic N2-fixing, *Dioscorea esculenta* L. Miyako Island, Japan	GCF_009176305.1			[57]
* Rhizobium mongolense* subsp. *loessense* CGMCC 1.3401^T^ (=DSM 21811^T^= LMG 21975^T^)		GCF_900099775.1			[11]
'*Rhizobium oryzihabitans'* M15^T^ (=JCM 32903^T^=ACCC 60121^T^)	Rice roots	GCF_010669145.1			[58]
* Rhizobium rhizophilum* 7209-2^T^ (=CGMCC 1.15691^T^=DSM 103161^T^)	Rhizosphere of *Brassica napus* L., Mongolia, PR China	GCF_004912145.1			[59]
* Rhizobium ruizarguesonis* UPM1133^T^ (=CECT 9542^T^=LMG 30526^T^)	Nodules of *Pisum sativum* plants grown on Ni-rich soils	GCF_012349115.1			[17, 18]
*'Rhizobium terrae'* NAU-18^T^ (=KCTC 62418^T^=CCTCC AB 2018075^T^)	Oil-contaminated soil, PR China	GCF_003425685.1			[60]
*** Trinickia ***					
*'Trinickia dabaoshanensis'* CCTCC M 209109^T^ (GIMN-1.004^T^=LMG 30479^T^)	Heavy-metal-tolerant, Dabaoshan mining area soil, PR China				[61]

## Minute 7. Recent relevant publications in genome-based taxonomy

**7.1.** Based on an extensive genomic and phenotypic analysis of the genus *Ensifer */* Sinorhizobium *, Fagorzi *et al.* [10] concluded that it is made of two distinct clades, with inter-clade genomic similarity under 85 % average nucleotide identity (ANI) and 90 % average amino acid identity (AAI). They presented a large body of biological evidence suggesting that these clades harbour significantly different organisms, with one containing symbiotic strains and the other non-symbiotic strains. Conveniently, the phylogenetic boundary puts the type species and strains of *Sinorhizobium* and *Ensifer* in each of these respective clades. This indicates that the genus could be separated (again) into the genera *Ensifer* and *Sinorhizobium *, but Fagorzi *et al.* stop short of making a formal taxonomic proposal, although their findings are supported by several other recent reports [5, 11]. The transfer of *Sinorhizobium* to *Ensifer,* proposed 20 years ago [12], was never accepted by the wider scientific community, as witnessed by the tenfold higher number of recent publications using the name *Sinorhizobium *. Resurrection of the distinction would make biological sense, as it would now separate symbiotic and non-symbiotic clades.

**7.2.** We discussed the report of Weisberg *et al.* [3], which mainly focuses on oncogenic plasmids of agrobacteria, including a large set of strains, introducing several interesting new methods of analysis, defining nine distinct plasmid groups, and having potential impacts on taxonomy of these bacteria. Advances in Ti/Ri plasmid analysis are significant, but the classification of the bacteria carrying these plasmids remains unclear. The polyphyletic group agrobacteria (tumorigenic and rhizogenic *Rhizobiaceae* species) is somehow considered as a taxonomic entity, without considering current evidence that biovar 2 belongs to the genus *Rhizobium* and biovar 3 to the genus *Allorhizobium *. It is clear that Ti plasmids are transmissible among several genera in the family *Rhizobiaceae *, even though transmission events remain rare. Known biovars of agrobacteria remain the main hosts of oncogenic plasmids, with evidence of correlation and stable coexistence between chromosomal backgrounds and Ti plasmids, likely explained by their co-evolution. In this respect it was also recently reported that Ti plasmids can be maintained in *Neorhizobium* species in nature [13]. In addition, Weisberg *et al.* [3] suggested that there is a new group of pathogenic agrobacteria that is apparently a sister species of biovar 2. It might be similar to the species *Rhizobium tumorigenes *, which should be checked when sequences are available.

**7.3.** Following Ormeño-Orrillo *et al.* [14], González *et al.* [15] reported a robust *Rhizobium* phylogenomic study using ribosomal protein genes that are recommended as phylomarkers. This serves as a framework to analyse the variation profile of the *Rhizobium* accessory genome and provides evidence on the lateral transfer of symbiotic plasmids, as shown before for the *tropici* group [16]. This report confirms the misclassification of a number of strains in the NCBI previously reported by several authors.

**7.4.** Hördt *et al.* [11] reported an extensive genome-based taxonomic revision of the *Alphaproteobacteria *, including rhizobia and agrobacteria and extending the family *Rhizobiaceae *, with relevant proposals at several levels (species, genus, family, order) that will presumably be soon submitted to the *International Journal of Systematic and Evolutionary Microbiology* (IJSEM) for validation.

**7.5.** *Rhizobium ruizarguesonis* [17, 18] is within genospecies C of the *R. leguminosarum* species complex, so this is now the formal name for genospecies C. There are now five named species within the *R. leguminosarum* species complex (* R. leguminosarum *, *R. laguerreae *, *R. sophorae *, *R. ruizarguesonis* and *'R. indicum')* and a recent study indicates that there are more that could be named in future [19].

## Minute 8. Adjournment

The meeting was adjourned at 13.17 UTC on 6 July 2020. As usual, it was decided to continue the meeting online until 18 December 2020.

## Minute 9. Impact of the Nagoya Protocol on taxonomy

To propose a valid description of a new species name, the type strain needs to be made available freely to the scientific community. Rule 30 [20] of the International Code of Nomenclature of Prokaryotes requires that a viable culture of that strain must be deposited in at least two publicly accessible culture collections in two different countries from which subcultures must be available with no access restriction. Changing these rules has been regularly discussed among scientists in recent years, especially regarding the acceptance of genome sequence availability in replacement of the deposition of a live bacterial culture, referring to the description of taxa not based on isolates, e.g. based on metagenome-derived sequences, which are at the moment restricted to the *Candidatus* status. At the origin of this requirement of type strain availability for species description is the principle that taxonomy exists to inform biology. Microbiologists should have access to described bacterial cultures, and not only to their genome sequences, to study their biological properties towards increasing knowledge in fundamental research and devising applications of these organisms. Yet, the difficulty remains for a number of authors to describe new species due to genetic resources access regulations in their respective countries, where, in some cases, even the genome sequence cannot be published. Public international culture collections follow the rules of the Nagoya Protocol and acknowledge the sovereign rights and legislation of the country of origin. They can only accept deposits which are obtained in respect of the laws and regulation of the country of origin. Thus, the certificate of deposit can only be issued if type strains are available without restriction, at least for research purposes. In Brazil, for instance, the law allows deposit of strains in collections abroad. However, the law requires foreign researchers to always have an official partnership with a Brazilian institution and Brazilian scientists to perform research on genetic materials from Brazil. In practice, this national regulation requires foreign researchers to sign material transfer agreements (MTAs) or other contracts with Brazilian institutions, which is already considered as a restriction on the distribution of type strains by the ICSP. In several countries, such as Algeria, strains cannot be deposited without restrictions on their distribution, which prevents any new species description in this country. This, of course, is an issue relevant to the taxonomy of all microorganisms, not specifically to those bacteria within the scope of this Subcommittee. The Subcommittee agreed that a solution to the problem needs to be worked out at the level of the national authorities whose rules and regulations are creating problems for their own researchers. It is the responsibility of the latter to explain the rules better to their national authorities and give them a better insight into the situation. If the authorities prohibit deposit of a type strain, then they should be made aware that no new bacteria from their country can be described. This is a loss for the local scientists as well as for science worldwide. If biodiversity is not described and made available beyond national borders, it will not be used and cannot lead to local benefits from use by international partners. All the documentation that public culture collections require from their customers in the framework of the Nagoya Protocol is precisely to ensure that the country of origin is acknowledged and any benefits can be equitably shared. Although in some particular situations some species can indeed be endemic to a single country, most bacterial taxa are likely to be found more widely if efforts are made to look for them. It must be emphasized that the endemic character of a species can be validated and further reinforced for the country of origin only by allowing its comparison (through its type strain) with future descriptions internationally. In the time course of this discussion, the Subcommittee became aware of a working group at the ICSP Executive Board level on type strain accessibility and MTAs attached to type strains. Soon an Editorial should be published in the IJSEM to remind readers what the purpose of a type strain is, and to make updated recommendations (ICSP Executive Board, 29 October 2020, minute 8; www.the-icsp.org/reports).

## Minute 10. Closing

The online phase of this meeting was closed on 18 December 2020.
